# Factors Related to Diabetes Mellitus in the Middle-Aged and Over in Taiwan

**DOI:** 10.3390/healthcare8030242

**Published:** 2020-07-29

**Authors:** Chia-Chun Liang, Wei-Chung Hsu, Yao-Te Tsai, Shao-Jen Weng, Shih-Chia Liu, Cheng-Hsiang Lin

**Affiliations:** 1 Department of Radiation Oncology, Chung-Kang Branch, Cheng-Ching General Hospital, Taichung 40764, Taiwan; wdataul2003@gmail.com (C.-C.L.); b751106@yahoo.com.tw (W.-C.H.); 2 Department of Occupational Therapy, Asia University, Taichung 41354, Taiwan; 3 Department of International Business, Feng Chia University, Taichung 40723, Taiwan; yaottsai@fcu.edu.tw; 4 Department of Industrial Engineering and Enterprise Information, Tunghai University, Taichung 40704, Taiwan; sjweng@thu.edu.tw; 5 Healthcare Systems Consortium, Tunghai University, Taichung 40704, Taiwan; 6 Department of Statistics, Tunghai University, Taichung 40704, Taiwan

**Keywords:** longitudinal data, left censoring, interval censoring, diabetes mellitus, elderly

## Abstract

*Study Objective*: to investigate the factors related to diabetes mellitus in the middle-aged and over in Taiwan. Method: data from seven surveys (in 1989–2011) from the “Taiwan Longitudinal Study on Aging” (TLSA), among cohort B (above the age 60 in 1989), cohort A (aged 50–66 in 1996), and cohort C (aged 50–66 in 2003), were analyzed by the interval-censored Cox model. Results: in the early aging stage (aged 60–64), diabetes mellitus prevalence among the same age appeared the lowest in cohort B, followed by cohort A; cohort C reveals the highest than the young generation. Moreover, suffering from hypertension and kidney diseases are closely related to diabetes mellitus, with the diabetes mellitus suffering hazard ratio of 2.53 (95%: 2.35, 2.73) and 1.26 (95%: 1.11, 1.44) times, respectively. For people with fair and poor self-rated health, the risk of suffering from diabetes mellitus is 1.16 (95%: 1.07, 1.27) and 1.50 (95%: 1.35, 1.67) times compared to people with good self-rated health, respectively. *Conclusions*: in this study, it is considered that an advanced interval censoring model analysis could more accurately grasp the characteristics of factors in people who are middle-aged and over suffering from diabetes mellitus in Taiwan.

## 1. Introduction

The top ten leading causes of death in Taiwan in 2018 accounted for 77.5% of all deaths that year. They were sequenced as cancer, heart diseases, pneumonia, cerebral vascular diseases, diabetes mellitus, accident injuries, chronic lower respiratory diseases, hypertensive diseases, nephritis, nephrotic syndrome and nephropathy, and chronic liver diseases and liver cirrhosis. Cancer was continuously ranked on top, while diabetes mellitus was ranked fifth [1]. Among the ten leading causes of death, cerebral vascular diseases, cardiovascular disorder, and nephropathy were closely related to diabetes mellitus.

According to Taiwan Health Promotion Administration (THPA) statistics, there were more than 2 million diabetes mellitus patients in Taiwan and the number continuously grew 25,000 per year. Long-term, improper glycemic control could easily result in complications that affects health and medical payments. Meanwhile, diabetes mellitus was also an important risk factor in coronary heart diseases and stroke [2,3,4,5,6]. According to national health insurance medical statistics [7], the number of visits, with health insurance, for people with diabetes mellitus in Taiwan was about 2.1 million in 2018. This was about 224.2 times the number of deaths in the year, in which people above 65 appeared the highest (30,331 people) on the visit rate (per 100 thousand population—about 3.4 times the total average). On the other hand, the average health insurance medical expense for diabetes mellitus was 11,570 points per person in 2018. Although it was not the disease with the highest medical expenses, it could not be ignored when medical payments for related complications were included. Relevant research also mentioned that life loss and economic loss of diabetes mellitus were not large when compared to malignant tumors and accidental injuries, but the loss exceeded malignant tumors when the complications of metabolic disease syndromes, e.g., stroke, heart diseases, and kidney diseases, were taken into account [8].

Furthermore, diabetes mellitus, the major chronic disease of the aged, could easily result in complications. According to a health promotion statistics report announced by the Taiwan Health Promotion Administration (THPA), diabetes mellitus prevalence of people above 65 in 2015–2018 was 26.5%. In comparison with younger people, the prevalence, above 65, was 2.76 times of those aged 40–64 [9]. Moreover, relevant research also revealed that the standardized mortality of diabetes mellitus showed increasing ages [10]. Such growth is related to the aging society in Taiwan. According to the population estimation of the Republic of China (2018–2065) [11], Taiwan became an aged society (above 14%) in 2018, and is estimated to become a super-aged society (over 20%) in 2026. The aging speed would exceed advanced countries, such as the USA and Japan. In this case, the effect of major diseases related to the aged, on individuals, families, and society, could not be neglected.

Human health conditions are developed as a result of suffering from diseases (and then, eventually, dying). In other words, suffering from a disease is the beginning of health degradation. In today’s aging society, the health conditions of the aged are a concerning focus, in which chronic diseases are the key factors in activity functions and self-rated health [12]. Depression and disability conditions of diabetes mellitus patients are key factors in self-rated health [13]. Cardiovascular diseases presented high correlations with disability [14]. Previous research has also looked at the key factors in stroke and diabetes mellitus of the aged in Taiwan [15,16]. Some research also discussed the effects of chronic disease, physical functions, and lifestyles of the middle-aged, and health changes of aged people [17,18,19,20,21,22,23,24,25]. Chiu et al. [26] discussed the correlation between BMI (Body Mass Index) changes and diabetes mellitus of the aged in Taiwan. Tsai and Lee [27] pointed out the key factors of weight, betel nut chewing, IADL (Instrumental Activity of Daily Living), exercise, hypertension, heart disease, kidney disease, drinking, and depression conditions in diabetes mellitus of the aged. Liang et al. [28] investigated the disparity in the healthy life expectancy of the elderly with hypertension and diabetes mellitus.

“Event history analysis” has become a trend in the fields of population, public health, epidemiology, and social science in past years, while survival analysis is popular in biomedicine, and it is called reliability analysis in the industry. In terms of survival analysis, death is the observed event, while the history of the disease occurrence of the research objects is studied in disease development. In addition to survival and disease development process, “event” could be any issue worthy of concern, e.g., marriage and birth. When longitudinal data were used for the so-called event history analysis, it was merely right censoring research. In fact, the significance of left censoring and interval censoring of survey data is usually unnoticed. For instance, when a senior high school student is asked about the time distribution for the beginning of smoking, he/she might answer, “I smoke, but I forgot the first time I smoked.” The event of smoking occurred before the survey, while the actual time was unknown; it was the situation of left censoring. The other senior high school student might answer, “I never smoke”. The interviewed age would be the right censoring time. Interval censoring referred to the time of an event appearing in a certain interval, and the actual time could not be observed. For instance, a patient with a malignant tumor had physical checks at a constant time and discovered cancer cell metastasis. The time for metastasis appeared in the interval between two time checks. A machine in a factory was regularly examined. At a specific time point, the machine was out of order. The breakdown time (failure time) appeared in an interval. Moreover, chronic diseases, e.g., diabetes mellitus, appeared in between two time checks that the occurrence of the disease appeared in the interval.

In sum, longitudinal data, right censoring of the aged in Taiwan used to be studied. However, this study, focusing on the aged suffering from diabetes mellitus, intended to consider the complete interval-censoring model. According to the 22-year “Taiwan Longitudinal Study on Aging” (TLSA) data, provided by THPA, seven surveys were preceded in 1989–2011. Three generations of the cohort B (aged above 60 in 1989), cohort A (aged 50–66 in 1996), and cohort C (aged 50–56 in 2003) were selected as the data source. Section 2 presents the data collection and interval-censored cox model. In Section 3, the proposed method is applied to analyze (i) the difference of diabetes mellitus morbidity risk among the aged in different cohorts, and was compared; and (ii) the factors in the aged in different cohorts suffering from diabetes mellitus. Lastly, conclusions and limitations are shown in Section 4.

## 2. Materials and Methods

### 2.1. Data

“Taiwan Longitudinal Study on Aging” (TLSA) conducted by the Taiwan Health Promotion Administration (THPA) was utilized as the data source. The data were technically cooperated by the former Department of Health Family Project Institute and University of Michigan Population Research Center and Institute of Elders, and referred to the questionnaire design of relevant research in the USA and Japan. The first survey was preceded in April–June 1989, aiming to establish the basic database of health norms and living behaviors of the aged in Taiwan. In addition to demographic variables, variables related to the aged, including health conditions, family and life conditions, economic conditions, leisure and entertainment model, and social participation, were covered for the reference to make relevant health and welfare policies. The survey first took household registration population, aged above 60 in 1988, in 331 townships (not including mountain villages) in Taiwan as the sampling population (aged population), and 4412 samples were abstracted with stratified multi-level random sampling. Moreover, 4049 samples completed the interview in 1989, with the visit rate of 91.8%. After the completion of the baseline survey, the follow-up interview was continued every 3–4 years (in 1993, 1996, 1999, 2003, 2007, and 2011).

Furthermore, the interview was preceded in 1996 and 2003 to establish the database of the middle-aged and aged people, aged above 50 in Taiwan as the basis for the follow-up long-term research data. With a supplementary sample multiple cohort study design, the survived generation samples were continuously tracked, and the generation samples, aged 50–66, and the new generation samples, aged 50–56, were sampled for the survey. It aimed to analyze the health conditions and life needs of middle-aged and aged people aged above 50 in Taiwan for cross-sectional representativeness and longitudinal comparison.

The judgment of diabetes mellitus morbidity in this study was based on the case’s self-description and confirmed by the physician. The measurement of incidence time referred to the actual age of the case at the time.

### 2.2. Statistical Analysis

Regarding the morbidity of diabetes mellitus in TLSA, it was left censoring when the case suffered from diabetes mellitus in the baseline survey. When the case did not suffer from diabetes mellitus in the previous survey, but did in the next survey, it was interval censoring. The case not suffering from diabetes mellitus, until the end of the survey, was right censoring.

**Figure 1:**
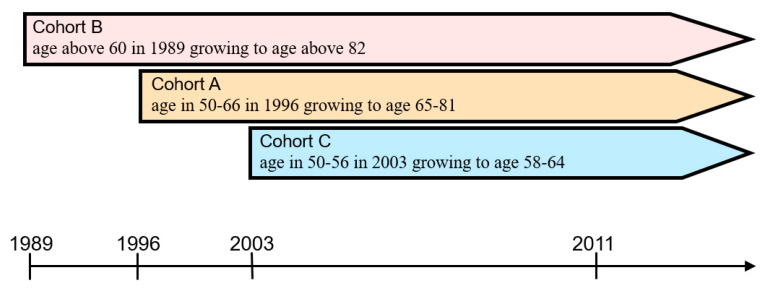
Surveyed samples of the middle-aged and over in Taiwan.

With the censoring characteristics of longitudinal data, TLSA was utilized as the data source for investigating the factors in diabetes mellitus of the aged in Taiwan and the generation difference. According to data from the seven surveys in 1989–2011, the interval-censored Cox model was used for analyzing the association between diabetes mellitus and its morbidity risk of the aged in Taiwan. The morbidity risks include demographic variables and physical and mental conditions, family environment, social participation, health behaviors, and chronic disease suffering situations. Regarding the discussion on different generations, cohort B—aged above 60 in 1989 (4049 completed samples) through 22 years, cohort A—aged 50–66 in 1996 (2462 completed samples) through 15 years, and cohort C—aged 50–56 in 2003 (1599 completed samples), through 8 years, were regarded as three different generations—aged, middle-aged, and young—from the viewpoint of the population life course. The above three cohorts were aged 82+ (cohort B, aged above 60 in 1989, aging to above 82), aged 65–81 (cohort A, aged 50–66 in 1996 and aging to 65–81), and aged 58–64 (cohort C, aged 50–56 in 2003, aging to 58–64) in 2011. The seven surveys showed the richest data of cohort B, followed by cohort A (5 surveys), and cohort C merely presented 3 surveys. Such data were regarded as random samples of surveys at a different time interval. After the analysis with the interval-censored Cox model, the probability of the middle-aged and over suffering from diabetes mellitus was constructed. The information constructed from the survey data in 1989–2011 is shown in Figure 1.

The probability of people aged 50–60 in cohort B in 2011 was constructed by the information from cohort A and cohort C; the information of those aged above 82 in cohort A was provided by cohort B, while the information of people aged above 65 in cohort C relied on the information of cohorts B and A. A probability of less than 0.05 is considered significant. All analyses were carried out using SAS version 9.4 (SAS Institute, Cary, NC, USA). The interval-censored Cox models were fitted using a SAS procedure, PROC ICPHREG.

## 3. Results

The characteristics and conditions of different cohorts at the baseline survey (1989 for cohort B, 1996 for cohort A, and 2003 for cohort C) were shown in Table 1 and Table 2. The characteristics of the samples at the baseline survey were explained as follows.

Males were more than females in all three cohorts (cohort B: 57.08%, cohort A: 51.50%, cohort C: 50.84%). Regarding the distribution of ethnic groups, Fukien appeared the most, Mainlanders was about one-quarter in cohort B, Hakka showed 15–17%, and aboriginals were merely 1.6–2.3%. In regards to education, most presented the education under elementary schools, and the proportion of illiteracy and elementary schools in cohorts B, A, and C were 41.58% vs. 39.57%, 25.38% vs. 50.24%, and 2.94% vs. 48.59%, respectively. About 16–35% samples did not have spouses; about 38% to half of the aged lived in urban areas; 80% of the aged in cohort B and cohort A showed adequate or more monthly expenses as the economic conditions (cohort B: 82.32%, cohort A: 80.84%, cohort C: 68.54%); and, about 40% of the aged participated in social activities.

Concerning the distribution of common complications related to chronic diseases and diabetes mellitus, the aged suffering from hypertension, heart diseases, and cataracts appeared 26.57%, 21.71%, and 14.44%, respectively, which was higher than those suffering from stroke and kidney diseases, 4.34% and 6.29%, respectively. Regarding the distribution of common complications related to chronic diseases and diabetes mellitus in cohort A, it was apparent on hypertension, 21.27%, while heart diseases, cataracts, stroke, and kidney diseases appeared below 10%. For cohort C, it was more obvious on hypertension, 19.21%, and heart diseases, cataract, stroke, and kidney diseases appeared below 10%.

Most of the aged presented good ADL at the baseline survey, more than 90%. More than 80% of the aged revealed favorable physical conditions (cohort B: 81.73%, cohort A: 94.19%, cohort C: 95.81%) and about 10–20% appeared to have depression conditions. In terms of self-rated health, the proportions of the aged who regarded themselves as being healthy in cohorts B, A, and C were 37.99%, 42.08%, and 56.41%, respectively.

The proportion of smoking in cohorts B, A, and C were 34.56%, 28.84%, and 26.95%, respectively. For drinking, the proportions were 21.19% (cohort B), 25.02% (cohort A), and 41.15% (cohort C), respectively. About 10% or less of the aged participated in betel nut chewing (cohort B: 5.42%, cohort A: 9.55%, cohort C: 9.69%). More than 90% of the aged engaged in outdoor activities in the previous half year.

Table 3 showed the results of the interval-censored Cox model. In addition to the analysis in various cohorts, the data of such cohorts were also integrated for analyses. Overall, the aged suffering from hypertension and kidney diseases showed higher risks of suffering from diabetes mellitus, about 2.53 times and 1.26 times, respectively. Furthermore, it was also discovered in the results that ADL and self-rated health were risk factors in the aged suffering from diabetes mellitus, where ones with fair and poor self-rated health presented the risk in suffering from diabetes mellitus 1.16 times and 1.50 times compared to those with good self-rated health, respectively. It was worth mentioning that ones with worse ADL appeared to have lower risks in suffering from diabetes mellitus, possibly because they required better care. On the other hand, it was also discovered that ones with good ADL did not necessarily show good health conditions. It, therefore, appeared a different direction from self-rated health.

**Figure 2:**
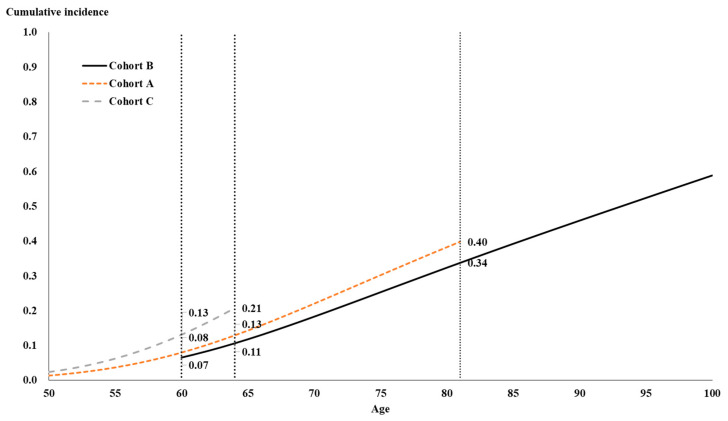
The cumulative incidence of diabetes mellitus by different cohorts.

Figure 2 showed the cumulative incidences of diabetes mellitus under the control of other variables (i.e., cumulative incidence function, CIF). The adjusted curve of variables was calculated by fixing the mean of other variables. Moreover, samples in cohorts B, A, and C experienced different length of survey times and were the cohort samples at different ages. Cohort B: aged above 60 in 1989, aging to above 82 in 2011; cohort A: aged 50–66 in 1996, aging to 65–81 in 2011; cohort C: aged 50–56 in 2003, aging to 58–64 in 2011). The age interval of such samples in various cohorts (cohort B: aged above 60; cohort A: aged 50–81; cohort C: aged 50–64) were further explained as below, where the overlapping age interval of three cohorts was the age group 60–64. After controlling the mean of various factors, the cumulative incidences of diabetes mellitus of samples in cohorts B, A, and C at the age of 60 appeared 0.07, 0.08, and 0.13, respectively, and 0.11, 0.13, and 0.21, respectively, at the age of 64, while it, respectively, showed 0.34 and 0.40 for cohorts B and A at the age of 81. The above results revealed that, under the same age, the cumulative incidence of a younger cohort suffering from diabetes mellitus was higher than the elder cohort.

**Figure 3:**
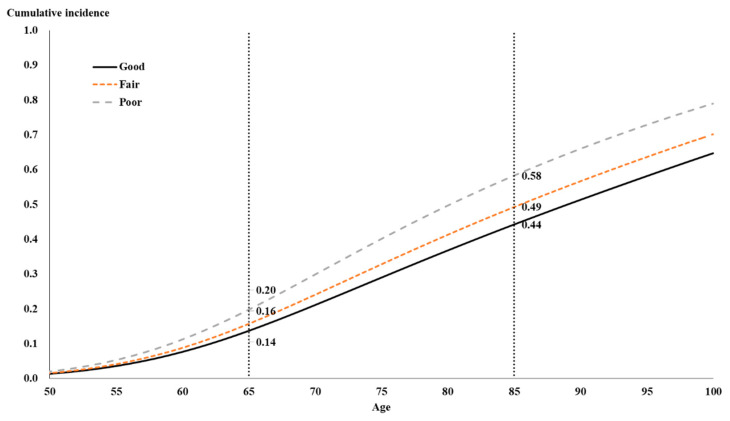
The cumulative incidences of diabetes mellitus by self-rated health.

Similarly, Figure 3 shows the overall trend of the cumulative incidence curves of diabetes mellitus of self-rated health, after controlling the mean of various factors. Ones with good, fair, and poor self-rated health revealed the cumulative incidences 0.14, 0.16, and 0.20, respectively, at the age of 65, and 0.44, 0.49, and 0.58, respectively, at the age of 85.

On the other hand, it was also estimated the probability of the aged in different cohorts suffering from diabetes mellitus at specific ages, Table 4. Overall, the probability of diabetes mellitus morbidity grew from 0.28% at the age of 50 to 1.48% at the age of 85, where the probability exceeded 1% at the age of 60, and then declined after the age of 76. In terms of cohort difference, cohort C was higher than cohort A at the age of 50–59, and the probability of cohort C exceeded 1% at the age of 56. In the overlapping age group, 60–64, of three cohorts, cohort B with the same age appeared to have the lowest probability of diabetes mellitus morbidity, followed by cohort A, and the younger generation of cohort C was the highest. At the age of 65–81, cohort A was higher than cohort B, without large differences. Besides, the probability of diabetes mellitus, morbidity of cohort B declined from the age of 77, while cohort A showed the age of 74.

## 4. Discussion

The cooperative research of National Taiwan University College of Public Health and Taiwan Health Promotion Administration made public the ranking of health hazard factors in Taiwan last year. The research integrated health databases in Taiwan, including nationally representative health surveys, cause-specific mortality from the National Death Registry, and relative risks from epidemiological studies and meta-analyses, to evaluate three major hazard factors of environment, behavior, and physiological metabolism. The results revealed that hyperglycemia was the most important hazard factor in death burden [29]. Moreover, according to the statistics of the National Health Insurance Administration, about 1.9 million diabetes mellitus patients saw doctors in 2015 with health insurance expenses of about 23.76 billion dollars, which was ranked the top three expenditures of health insurance. For this reason, the control of diabetes mellitus could reduce a huge amount of medical expenses; meanwhile, reducing the increasing speed of diabetes mellitus patients could reduce relevant health insurance expenses and resources. Apparently, it would be the prior issue to prevent and cure such a hazard factor.

Diabetes mellitus is a primary death disease, while long-term improper glycemic control could result in many complications. Among the ten leading causes of death, about half of the causes are related to diabetes mellitus complications. Diabetes mellitus patients would not show specific symptoms in an early invasion, but merely some uncomfortable feelings, which are not easily observed without checks. Diabetes mellitus is now a common disease and the morbidity situation is increasing with enhancing living standards, especially in middle-aged people aged 40–50.

It is not dreadful suffering from diabetes mellitus. However, diabetes mellitus patients need long-term glycemic control, and the factors of family and economy often result in bad glycemic control. In this case, complications derived from bad glycemic control, such as kidney dialysis, stroke, amputation, cataract, and heart diseases, are dreadful for diabetes mellitus patients and the family. What is more, diabetic retinopathy is the major cause of blindness of adults domestically. Under the increasing demands for long-term care, long-term care derived from diabetes mellitus would be a heavy burden for the government, the patient, and the family.

## 5. Conclusions

The results in this study also revealed that the aged in the younger cohort appeared to have a higher probability of diabetes mellitus morbidity at the same age as the older cohort. Besides, the probability of diabetes mellitus morbidity, in the age group of 60–64, also revealed a higher probability in the younger cohort, where the probability of cohort C at the age of 64 exceeded 2%. Although metabolic disease syndromes of stroke, heart diseases, and kidney diseases are with no significant risks of suffering from diabetes mellitus, these symptoms should not be ignored. Patients would realize it after suffering from acute cardiovascular diseases or other complications. It, therefore, is a major disease that should not be neglected. The research results also revealed higher risks of diabetes mellitus for the aged suffering from hypertension or kidney diseases; hypertension was especially obvious. In addition to objectively reflecting the health situations, self-rated health could also be the risk reference for suffering from diabetes mellitus.

On the other hand, measurements of height and weight in TLSA were proceeded with self-reports that being overweight and obesity might be deviated. For this reason, they were not included in the BMI anthropometric measurement. It was regarded as a limitation of this study.

In consideration of the urgency of diabetes mellitus control, as well as the effect of high-speed aging in Taiwan, the prevention of diseases, in addition to the continuous promotion of preventive health service, is the most economical way to have the aged be independent in daily life, rather than the treatment after the invasion. Different from the past research, censoring mechanisms were covered in the research model and the interval-censored Cox model was utilized for analyzing relevant factors in diabetes mellitus of the aged in Taiwan. Moreover, cohort-tracking data were discussed—that the analysis results could be the reference for relevant units coping with the situation, and accurately grasp the characteristics of factors in the aged suffering from diabetes mellitus to further make relevant prevention strategies and promotion. The relevant analyses could be promoted to the application to other diseases.

## Figures and Tables

**Table 1:** Characteristics and status of different cohorts at the baseline survey.

Characteristics and Status		Cohort B (*n* = 4049)		Cohort A (*n* = 2462)		Cohort C (*n* = 1599)	
		*n*	%	*n*	%	*n*	%
Gender	Female	1738	42.92	1194	48.50	786	49.16
	Male	2311	57.08	1268	51.50	813	50.84
Ethnicity	Fukien	2451	60.92	1808	73.47	1200	75.04
	Hakka	603	14.99	425	17.27	278	17.39
	Mainlander	900	22.37	189	7.68	85	5.32
	Aboriginal	69	1.72	39	1.58	36	2.25
Education	Illiteracy	1676	41.58	625	25.38	47	2.94
	Elementary school	1595	39.57	1237	50.24	777	48.59
	Junior high school	327	8.11	251	10.19	231	14.45
	Senior high school	433	10.74	349	14.18	544	34.02
Spouse	Yes	2618	64.69	2061	83.71	1327	82.99
	No	1429	35.31	401	16.29	272	17.01
Residential location	City	1917	47.35	935	38.33	777	48.81
	Town	726	17.93	575	23.58	367	23.05
	Countryside	1406	34.72	929	38.09	448	28.14
Economic status	Good	1683	43.20	822	35.16	577	36.76
	Fair	1524	39.12	1068	45.68	499	31.78
	Poor	689	17.68	448	19.16	494	31.46
Social activity	Yes	1564	38.63	978	39.79	649	40.59
	No	2485	61.37	1480	60.21	950	59.41

**Table 2:** Characteristics and status of different cohorts at the baseline survey (continued).

Characteristics and Status		Cohort B (*n* = 4049)		Cohort A (*n* = 2462)		Cohort C (*n* = 1599)	
		*n*	%	*n*	%	*n*	%
Hypertension	No	2960	73.43	1906	78.73	1283	80.79
	Yes	1071	26.57	515	21.27	305	19.21
Heart disease	No	3159	78.29	2197	90.90	1494	93.84
	Yes	876	21.71	220	9.10	98	6.16
Cataract	No	3460	85.56	2247	91.34	1554	97.43
	Yes	584	14.44	213	8.66	41	2.57
Stroke	No	3856	95.66	2392	97.35	1570	98.25
	Yes	175	4.34	65	2.65	28	1.75
Kidney disease	No	3786	93.71	1313	94.22	1485	93.28
	Yes	254	6.29	142	5.78	107	6.72
ADL ^a^	Good	3801	94.04	2425	98.50	1590	99.44
	Fair	93	2.30	20	0.81	3	0.19
	Poor	148	3.66	17	0.69	6	0.38
Physical function ^b^	Good	3307	81.73	2319	94.19	1532	95.81
	Fair	510	12.61	102	4.14	48	3.00
	Poor	229	5.66	41	1.67	19	1.19
Depression ^c^	No	3036	77.91	1943	82.86	1397	88.98
	Yes	861	22.09	402	17.14	173	11.02
Self-rated health	Good	1526	37.99	988	42.08	902	56.41
	Fair	1491	37.12	783	33.35	443	27.70
	Poor	1000	24.89	577	24.57	254	15.88
Smoking	No	2649	65.44	1752	71.16	1168	73.05
	Yes	1399	34.56	710	28.84	431	26.95
Alcohol	No	3191	78.81	1846	74.98	941	58.85
	Yes	858	21.19	616	25.02	658	41.15
Betel nut chewing	No	3825	94.58	2227	90.45	1444	90.31
	Yes	219	5.42	235	9.55	155	9.69
Outdoor activity	Yes	3906	96.47	2442	99.19	1593	99.62
	No	143	3.53	20	0.81	6	0.38

Notes: ^a^ ADLs (Activities of Daily Living) referred to the difficulty of a case in taking a bath, putting on/taking off clothes, eating, standing up from the bed or sitting on a chair, walking indoors, and going to the toilet. The lower difficulty revealed the better ADL; ^b^ Physical functions referred to a case being able to walk to the second or third floor, walk 200–300 m, do heavy work around the house, lift something weighting 20 Taiwanese kilograms, crouch, raise hands above the head, and take or turn something with fingers. The higher value revealed better physical functions. ^c^ Depression was divided according to depression scale (CES-D), including bad appetite, bad mood, not doing well, not sleeping well, feeling happy, feeling lonely, regarding people being unfriendly, feelings of enjoying life, feeling sad, and low spirit. They were scored in 0–30, and the higher score, above, or equal to 10, was classified into depression conditions; otherwise, they were classified as not being depressed.

**Table 3:** Hazard ratio (HR) between variables and diabetes mellitus using the interval-censored Cox model.

Variables		Overall		Cohort B		Cohort A		Cohort C	
		HR	95% CI	HR	95% CI	HR	95% CI	HR	95% CI
Hypertension	No	1		1		1		1	
	Yes	2.53 ***	2.35–2.73	3.56 ***	3.18–3.98	1.79 ***	1.49–2.14	2.45 ***	1.86–3.23
Heart disease	No	1		1		1		1	
	Yes	1.10	0.99–1.21	1.06	0.94–1.21	1.20	0.94–1.54	0.98	0.62–1.54
Cataract	No	1		1		1		1	
	Yes	1.04	0.93–1.16	1.04	0.90–1.20	1.15	0.89–1.47	1.52	0.82–2.81
Stroke	No	1		1		1		1	
	Yes	1.18	0.97–1.44	1.07	0.80–1.43	1.26	0.78–2.03	1.12	0.52–2.41
Kidney disease	No	1		1		1		1	
	Yes	1.26 ***	1.11–1.44	1.50 ***	1.24–1.81	1.07	0.78–1.48	0.88	0.54–1.43
ADL	Good	1		1		1		1	
	Fair	0.68 *	0.48–0.98	0.65	0.42–1.02	0.54	0.17–1.76	1.40	0.24–8.23
	Poor	0.53 **	0.34–0.83	0.63	0.38–1.05	0.56	0.10–3.21	1.38	0.14–13.35
Physical function	Good	1		1		1		1	
	Fair	1.02	0.89–1.16	0.86	0.73–1.02	1.37	0.98–1.91	1.10	0.57–2.15
	Poor	1.14	0.87–1.48	0.78	0.55–1.11	2.07	0.78–5.51	2.46	0.91–6.64
Depression	No	1		1		1		1	
	Yes	1.03	0.93–1.14	0.97	0.84–1.12	0.94	0.75–1.19	1.27	0.86–1.87
Self-rated health	Good	1		1		1		1	
	Fair	1.16 ***	1.07–1.27	1.20 **	1.06–1.37	1.04	0.85–1.27	1.59 **	1.18–2.16
	Poor	1.50 ***	1.35–1.67	1.51 ***	1.27–1.79	1.42 **	1.13–1.79	1.88 **	1.26–2.81

Notes: * *p* < 0.05; ** *p* < 0.01; *** *p* < 0.001. Control of gender, ethnic group, education, smoking, betel nut chewing, outdoor activity, spouse, residential type, economic conditions, and social activity were included in the model.

**Table 4:** Probability of diabetes mellitus morbidity at the age of 50–85.

Age	Overall	Cohort B	Cohort A	Cohort C
50	0.0028		0.0029	0.0047
51	0.0033		0.0034	0.0056
52	0.0039		0.0040	0.0066
53	0.0046		0.0046	0.0076
54	0.0052		0.0053	0.0087
55	0.0060		0.0060	0.0099
56	0.0068		0.0068	0.0112
57	0.0076		0.0077	0.0124
58	0.0084		0.0085	0.0137
59	0.0093		0.0094	0.0150
60	0.0101	0.0084	0.0102	0.0163
61	0.0110	0.0092	0.0111	0.0175
62	0.0118	0.0099	0.0119	0.0186
63	0.0126	0.0105	0.0127	0.0197
64	0.0133	0.0112	0.0134	0.0206
65	0.0139	0.0117	0.0141	
66	0.0145	0.0123	0.0146	
67	0.0150	0.0127	0.0151	
68	0.0154	0.0131	0.0155	
69	0.0157	0.0134	0.0158	
70	0.0159	0.0136	0.0160	
71	0.0161	0.0138	0.0162	
72	0.0162	0.0140	0.0163	
73	0.0162	0.0141	0.0164	
74	0.0163	0.0141	0.0164	
75	0.0162	0.0142	0.0163	
76	0.0162	0.0142	0.0163	
77	0.0161	0.0142	0.0162	
78	0.0160	0.0141	0.0161	
79	0.0158	0.0141	0.0159	
80	0.0157	0.0140	0.0158	
81	0.0155	0.0139	0.0156	
82	0.0153	0.0138		
83	0.0152	0.0137		
84	0.0150	0.0136		
85	0.0148	0.0135		

## References

[1] Statistics Division, Ministry of Health and Welfare, ROC (Taiwan) (2020). 2018 Cause of Death Statistics.

[2] Assmann G., Schulte H. (1989). Diabetes mellitus and hypertension in the elderly: Concomitant hyperlipidemia and coronary heart disease risk. Am. J. Cardiol..

[3] Chan P., Pan W.H. (1995). Coagulation activation in type 2 diabetes mellitus: The higher coronary risk of female diabetic patients. Diabet. Med..

[4] Cleland S.J., Petrie J.R., Ueda S., Elliott H.L., Connell J.M. (1998). Insulin as a vascular hormone: Implications for the pathophysiology of cardiovascular disease. Clin. Exp. Pharmacol. Physiol..

[5] Kaplan N.M. (1989). The deadly quartet. Upper-body obesity, glucose intolerance, hypertriglyceridemia, and hypertension. Arch. Intern. Med..

[6] Chen K.T., Chen C.J., Fuh M.M., Narayan K.M. (1999). Cause of death and associated factors among patients with non-insulin-dependent diabetes mellitus in Taipei, Taiwan. Diabetes Res. Clin. Pract..

[7] National Health Insurance Administration, Ministry of Health and Welfare, ROC (Taiwan) (2020). National Health Insurance Annual Statistical Report 2018.

[8] Lin C.H., Liu S.C., Liu G.W. (2015). The effects of trends in mortality due to the leading causes of death on potential lost life and economic loss in Taiwan. Taiwan J. Public Health.

[9] Health Promotion Administration, Ministry of Health and Welfare, ROC (Taiwan) (2020). Statistics of Health Promotion 2018.

[10] Tseng C.H., Chong C.K., Heng L.T., Tseng C.P., Tai T.Y. (2000). The incidence of type 2 diabetes mellitus in Taiwan. Diabetes Res. Clin. Pract..

[11] National Development Council, ROC (Taiwan) (2018). Population Projections for ROC (Taiwan): 2018-2065.

[12] Molarius A., Janson S. (2002). Self-rated, Chronic disease, and symptoms among middle-aged and elderly men and women. J. Clin. Epidemiol..

[13] Badawi G., Gariépy G., Pagé V., Schmitz N. (2012). Indicators of self-rated health in the Canadian population with diabetes. Diabetic Med..

[14] Anderson R.T., James M.K., Miller M.E., Worley A.S., Longino C.F. (1998). The timing of change: Patterns in transitions in functional status among elderly persons. J. Gerontol. Ser. B Psychol. Sci. Soc. Sci..

[15] Lee T.K., Huang Z.S., Ng S.K., Chan K.W., Wang Y.S., Liu H.W., Lee J.J. (1995). Impact of alcohol consumption and cigarette smoking on stroke among the elderly in Taiwan. Stroke.

[16] Tsai A.C., Chi S.H., Wang J.Y. (2015). Association of perceived stress with depressive symptoms in older Taiwanese: Results of a population-based study. Geriatr. Gerontol. Int..

[17] Wang S.H., Wang J.Y. (2013). The decline in physical and mental health among older chronic stroke patients in Taiwan. Taiwan J. Public Health.

[18] Chiu H.P., Tsai A.C., Wang J.Y. (2014). Combined effect of body mass index and physical activity on the decline in walking ability amongst older Taiwanese. Taiwan J. Public Health.

[19] Chen T.J., Wang J.Y. (2016). Association between reading and cognitive decline in older Taiwanese people. Taiwan J. Public Health.

[20] Chi S.H., Wang J.Y., Tsai A.C. (2016). Combined association of leisure-time physical activity and fruit and vegetable consumption with depressive symptoms in older Taiwanese: Results of a national cohort study. Geriatr. Gerontol. Int..

[21] Hsu W.C., Chen L.C., Wang J.Y. (2017). Predicting emerging care-need with simple functional indicators-findings from a national cohort study in Taiwan. Geriatr. Gerontol. Int..

[22] Tsai A.C., Liou J.C., Chang M.C. (2007). Interview to study the determinants of hypertension in older adults in Taiwan: A population based cross-sectional survey. Asia Pac. J. Clin. Nutr..

[23] Wang J.Y., Tsai A.C. (2013). The short-form mini-nutritional assessment is as effective as the full-mini nutritional assessment in predicting follow-up 4-year mortality in elderly Taiwanese. J. Nutr. Health Aging.

[24] Gómez-Pimienta E., González-Castro T.B., Fresan A., Juárez-Rojop I.E., Martínez-López M.C., Barjau-Madrigal H.A., Ramírez-González I.R., Martínez-Villaseñor E., Rodríguez-Sánchez E., Villar-Soto M. (2019). Decreased quality of life in individuals with type 2 diabetes mellitus is associated with emotional distress. Int. J. Environ. Res. Public Health.

[25] Pizzol D., Smith L., Koyanagi A., Stubbs B., Grabovac I., Jackson S.E., Veronese N. (2019). Do older people with diabetes meet the recommended weekly physical activity targets? An analysis of objective physical activity data. Int. J. Environ. Res. Public Health.

[26] Chiu C.J., Li S.L., Wu C.H., Du Y.F. (2015). BMI trajectories as a harbinger of pre-diabetes or underdiagnosed diabetes: An 18-year retrospective cohort study in Taiwan. J. Gen. Intern. Med..

[27] Tsai A.C., Lee S.H. (2015). Determinants of new-onset diabetes in older adults—Results of a national cohort study. Clin. Nutr..

[28] Liang C.C., Hsu W.C., Tsai Y.T., Weng S.J., Yang H.P., Liu S.C. (2020). Healthy life expectancies by the effects of hypertension and diabetes for the middle aged and over in Taiwan. Int. J. Environ. Res. Public Health.

[29] Lo W.C., Ku C.C., Chiou S.T., Chan C.C., Chen C.L., Lai M.S., Lin H.H. (2017). Adult mortality of diseases and injuries attributable to selected metabolic, lifestyle, environmental, and infectious risk factors in Taiwan: A comparative risk assessment. Popul. Health Metr..

